# Statistical estimates of absenteeism attributable to seasonal and pandemic influenza from the Canadian Labour Force Survey

**DOI:** 10.1186/1471-2334-11-90

**Published:** 2011-04-12

**Authors:** Dena L Schanzer, Hui Zheng, Jason Gilmore

**Affiliations:** 1 Infectious Disease and Prevention and Control Branch, Public Health Agency of Canada, Ottawa, Ontario, Canada; 2 Statistics Canada, Ottawa, Ontario, Canada

## Abstract

**Background:**

As many respiratory viruses are responsible for influenza like symptoms, accurate measures of the disease burden are not available and estimates are generally based on statistical methods. The objective of this study was to estimate absenteeism rates and hours lost due to seasonal influenza and compare these estimates with estimates of absenteeism attributable to the two H1N1 pandemic waves that occurred in 2009.

**Methods:**

Key absenteeism variables were extracted from Statistics Canada's monthly labour force survey (LFS). Absenteeism and the proportion of hours lost due to own illness or disability were modelled as a function of trend, seasonality and proxy variables for influenza activity from 1998 to 2009.

**Results:**

Hours lost due to the H1N1/09 pandemic strain were elevated compared to seasonal influenza, accounting for a loss of 0.2% of potential hours worked annually. In comparison, an estimated 0.08% of hours worked annually were lost due to seasonal influenza illnesses. Absenteeism rates due to influenza were estimated at 12% per year for seasonal influenza over the 1997/98 to 2008/09 seasons, and 13% for the two H1N1/09 pandemic waves. Employees who took time off due to a seasonal influenza infection took an average of 14 hours off. For the pandemic strain, the average absence was 25 hours.

**Conclusions:**

This study confirms that absenteeism due to seasonal influenza has typically ranged from 5% to 20%, with higher rates associated with multiple circulating strains. Absenteeism rates for the 2009 pandemic were similar to those occurring for seasonal influenza. Employees took more time off due to the pandemic strain than was typical for seasonal influenza.

## Background

As many viruses can cause similar respiratory symptoms and laboratory confirmation is not routine, data specific to influenza is limited. Statistical estimates of the influenza burden identify a significant morbidity [1-5] and mortality [6-8] burden each year. Studies estimating the clinical attack rate and workplace absenteeism are limited, though workplace absenteeism is a significant component of the economic costs of influenza and uncertainty in absenteeism associated with influenza also contributes to the uncertainty of estimates of the economic burden [9,10]. Models used to calculate economic costs generally assume that 5% to15% of the population are affected with upper respiratory infections due to influenza viruses annually [11]. This range was obtained from estimates from cohort studies, and more recently the control arm of antiviral or vaccination effectiveness studies, many of which suggest that influenza affects 5-10% of the population each year [12], though some studies suggest rates may be as high as 26% [13]. The Canadian pandemic plan proposed a clinical attack rate of 15 to 35% over multiple waves of a pandemic strain [14]. While an influenza infection is generally associated with a high fever and cough, clinical symptoms associated with an influenza infection vary substantially, asymptomatic infections have been documented [15] and case ascertainment remains a challenge. Given the challenges in studying workplace absenteeism due to influenza infections, we aimed to adapt the statistical models used to estimate the morbidity and mortality burden to estimate workplace absenteeism rates and the proportion of potential hours worked that were lost due to seasonal and pandemic influenza from key variables from the Labour Force Survey, a monthly survey by Statistics Canada that provides timely estimates of employment and unemployment rates in Canada.

## Methods

The estimation of the number of deaths (or hospital admissions) attributable to influenza involves establishing a seasonal baseline for the weekly time-series of deaths (or respiratory admissions) to account for seasonality and secular trends, and then matching the weekly pattern of a proxy variable for influenza activity to the pattern of excess deaths (or admissions) [5,16,17]. Regression is used to jointly estimate the seasonal baseline, secular trends and the impact of influenza. Because peak influenza activity is concentrated over a relatively short period of time and because the timing of peak activity varies every year, this approach has been successful in detecting a relatively small burden. For example, 2% of annual deaths were attributed to influenza [6] using this approach. While the impact of other viruses has been simultaneously estimated, their impact was not always statistically significant and could be ignored without significantly altering the estimated burden attributed to influenza in an adult population.

### Data Sources

Monthly time series were extracted from Statistics Canada's Labour Force Survey (LFS) for key variables related to absenteeism rates and the proportion of potential hours worked that were lost due to illness or disability and employee characteristics [18]. The main objective of the LFS is to provide timely estimates of monthly employment levels and unemployment rates as an early indicator of the level of economic activity in Canada. Data collection for the LFS is carried out each month during the week following the LFS reference week, normally the week containing the 15th day of the month. Approximately 54,000 households are sampled each month. The sample is allocated to provinces and strata within provinces in a way that best meets the need for reliable estimates at various geographic levels. In each dwelling, information about all household members aged 15 and over is usually obtained from one knowledgeable household member. Each household remains in the survey for a period of six months. Of interest for this study was the reported number of hours that survey participants actually worked and the number of hours lost due to their own illness or disability. This data was obtained from Statistics Canada under their cost recovery program.

The weekly number of laboratory confirmations for influenza A and B were obtained from the *FluWatch *program, Public Health Agency of Canada (PHAC) [19]. From September 1995 to April 2009, specimens were submitted to participating laboratories by clinicians in the course of clinical care and patient management in inpatient, emergency room or outpatient settings, and by sentinel physicians participating in the national influenza surveillance program [20]. By April 26, 2009, the first cases of the pandemic strain (A/California/7/2009) were reported to PHAC [20] and testing rates initially increased sharply and then varied in response to public health needs over the pandemic period. During the 2009 H1N1 pandemic period, laboratory testing was used to identify hospital admissions associated with H1N1/09 infection, and admissions of patients with laboratory confirmation of the pandemic strain (A/California/7/2009) were reported to PHAC [20] by nine out of ten provinces and by the three territories. This series was used as a proxy for the level of influenza activity during the pandemic period.

### Statistical Analysis

Combining these data sources, the full study period available includes 15 seasons from September 1995 to February 2010. As the number of laboratory tests reported to *FluWatch *increased during the early years of the surveillance program, the analysis focused on the 11 influenza seasons from 1999/00-2008/09 with higher detection rates and the one pandemic season from May 2009 to April 2010.

Absenteeism rates (# of employed persons who were absent due to own illness or disability/# of employed persons) and the proportion of potential hours worked that were lost due to own illness or disability were modeled separately as a function of seasonality (month), secular trend, and the level of influenza activity corresponding to the reference week. The regression model was fit using SAS Enterprise Guide [21] PROC GENMOD with a binomial distribution, linear link function and dispersion parameter specified by:

where *HL *represents the hours lost due to own illness or disability and *PHW *the potential hours worked during the reference week for the category of interest (for example age group); the β_1 _parameters account for the baseline seasonality with monthly indicator variables (*Mon_m_*); the β_2 _parameters account for a general trend with indicator variables for each influenza season or flu year (*FY_y_*) starting in September; the β_3 _parameters account for hours lost due to influenza A infection, potentially varying by *FY *(*FluAr *is the number of weekly influenza A laboratory confirmations for the reference week, seasonal only); β_4 _accounts for the increase in hours lost due to influenza B infection (*fluBpp *is the percent of tests positive for influenza B); β_5 _accounts for any change in absenteeism behavior once the pandemic was announced that was not related to the level of influenza activity, that is an influenza infection (for example, staying home because of concern that a respiratory infection was due to the pandemic strain even though the employee would have otherwise reported for work and the infection was due to another respiratory virus); and β_6 _accounts for hours lost due to the H1N1/2009 pandemic strain (*Hospadmsr *is the number of laboratory confirmed hospital admissions in the reference week). Laboratory confirmed H1N1/2009 hospital admissions were used as a proxy for influenza activity during the pandemic period, as the number of laboratory confirmed hospital admissions was considered a better proxy for the level of influenza activity than the number of laboratory confirmed cases during the pandemic period. The *FluAr *variable (number of influenza A positive tests in the reference week) includes only seasonal influenza positive tests. The pandemic period was defined to start in May 2009 and continue until the end of the study period (*Pandemic2009*).

A similar model was fit for the absenteeism rates, with the proportion of employed persons who took time off work due to their own illness or disability in the reference week as the dependant variable. Absences and hours lost for care of others were considered for separate analysis, however, variation in these monthly time series were found to be minimal and not associated with influenza activity.

There are various models that have been used to estimate influenza-attributable events or excess mortality. All regression models include variables to explain the weekly or monthly seasonality and secular trends of the dependent variable and to account for the impact of influenza activity. The percent of tests positive for influenza is a convenient choice of proxy variable to account for the impact of influenza activity which easily normalises for differences in testing over time, while the use of the number of influenza A positive tests along with separate parameters for each season, in addition to providing a slightly better model fit, provided a redundancy that helped illustrate model robustness in previous work [1,16,17]. The effect of influenza B on workplace absenteeism (and hours lost) could not be estimated separately for each season due to limited statistical power and limited impact of influenza B on workplace absenteeism, so that *fluBpp *was used as one approach to account for the effects of influenza B in the model without over fitting. The weekly number of laboratory tests and confirmations for RSV, adenovirus and parainfluenza virus were also obtained from the *FluWatch *program and similarly included in alternative models to see if they might have a significant impact on absenteeism. Only the results for influenza B were statistically significant (at the 5% significant level), and hence considered for the final model described in the paper. Over fitting becomes a problem when parameter estimates start to vary widely and generally lose statistical significance. Omitting important explanatory variables can bias the remaining parameter estimates. A degree of consistency in the attribution to influenza from season to season (from estimating multiple β_3 _parameters) would provide face validity of model results and was one criterion considered in guiding model development.

A regression model approach facilitated the simultaneous estimation of the effects of influenza activity while controlling for other factors. Baseline rates were calculated from the fitted model by setting the proxy variables for influenza activity to zero. The difference between the model-predicted number of hours lost and the baseline is an estimate of the number of hours lost due to influenza. The excess hours lost (defined as actual less baseline) includes unexplained variation that may be due to (unknown) events unrelated to influenza. The unexplained variation will average out over each season due to the nature of the regression model. A linear link function was chosen to maintain a linear relationship between viral activity and absenteeism due to influenza. Confidence intervals for estimates of the proportion of hours lost due to influenza were calculated from the coefficient of variation of the corresponding parameter for the proxy variable for influenza activity. The dispersion parameter was included in the model estimation to account for additional variation due to events not captured by the choice of explanatory variables.

Annual absenteeism rates attributable to influenza were calculated by summing the predicted monthly absenteeism rates for each month within the indicated time period. Repeat influenza infections in one employee (and in different months) though rare, would be counted as two absences. The absenteeism rate was pro-rated to the full month based on the number of work days in the month with an adjustment for variation in the level of influenza throughout the month. The proportion of hours lost due to influenza was calculated by summing the estimated monthly hours lost due to influenza and the potential hours worked over the specified period and then dividing. The number of hours lost per absence due to influenza was calculated from the estimated number of hours lost due to influenza divided by the estimated number of absences attributed to influenza per season.

Differences in the annual estimates of absenteeism rates and the proportion of hours lost due to influenza were compared with the number and subtypes of the circulating strains from national year-end summary reports [20].

The proportion of potential hours worked that were lost due to influenza was also estimated by age group, sex, and employment characteristics (full-time/part-time; permanent/temporary; public/private sector; union coverage; and urban or rural residency) in separate regression models. As hours lost for the care of others had very little seasonality and the limited seasonal variation that was present was not associated with influenza activity, model estimates were not produced for the care of others from this data set and absenteeism due to influenza for the care of others could be considered negligible.

### Ethical Statement

This study was conducted in accordance with the principles expressed in the Declaration of Helsinki. Data provided by Statistics Canada were collected under the Statistics Canada Act and are available to the public through their cost recovery program. Data provided by the Public Health Agency of Canada were collected under the Public Health Agency of Canada Act and were used in agreement with policy and regulations related to the publication of information related to public health. Identifying information was not available to this study. Hence, ethics approval was not required.

## Results

An estimated 13% of employed persons in Canada took time off from work as a result of their own illness associated with the H1N1/2009 pandemic strain. Absenteeism rates for seasonal influenza averaged 12% over the 1997/98 to 2008/09 seasons. Typically 3% of potential hours worked are lost due to the employee's own illness or disability annually, though this figure varies with age and other employment characteristics. An average of 0.08% (95% CI: 0.06-0.10) of hours worked were lost annually due to seasonal influenza, while the proportion of potential hours worked that were lost due to influenza over the two pandemic waves was 0.19% (95% CI: 0.15-0.23) when pro-rated to an annual bases for comparison (Table 1). Seasonal influenza accounted for 3% of the hours lost annually, while the pandemic strain accounted for 6%. Absenteeism attributable to pandemic strain was highest in the months of October and November of 2009 at 3.3% and 6.7%, respectively.

**Table 1:** Absenteeism and percent of hours lost due to own illness or disability attributed to influenza in employed persons 15 years of age and older

Season/Wave	Length of Period	% of Hours Worked that were Lost Due to Own Illness or Disability	% of Potential Hours Worked that were Lost Due to Own Illness and Attributed to Influenza^1^	% of Hours Lost that were Attributable to Influenza	Estimated % of Employees Absent due to Influenza per Period (Wave/Season)^1^
Seasonal	Annual (12 months)	2.9%	0.08%	3%	11.5%
H1N1/09	Pro-rated to annual (May09-April10)	3.1%	0.19%	6%	13.4%
H1N1/09					
Spring	4 months (May-Aug09)	2.8%	0.12%	4%	2.9%
Fall	4 months (Sept-Dec09)	3.2%	0.47%	15%	10.5%
					
Oct-09	1 month	3.2%	0.59%^2^	18%	3.3%
Nov-09	1 month	3.9%	1.25%^2^	32%	6.7%^2^
Dec-09	1 month	3.0%	0.05%	2%	0.3%

^1 ^The attribution to influenza was estimated on an annual basis, as described in the methods sections. Monthly estimates were calculated as the model-predicted estimate less estimated baseline and agree with the influenza-attributed time series in Figure 4.

^2 ^The number of hours lost or number of absences reported for the indicated reference weeks were significantly above the estimated baseline (95% confidence level). The excess for these months is not shown in this table, though can be seen in Figures 4 and 5.

### Annual Estimates

**Figure 1:**
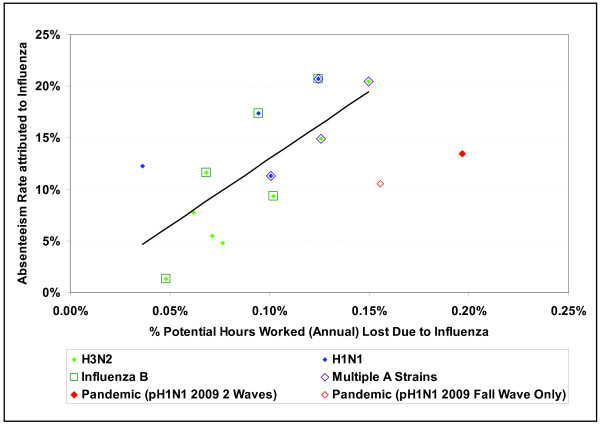
**Workplace absenteeism attributed to influenza: seasonal, 1997/98-2008/09, and pandemic 2009**. Influenza seasons differed by predominant subtype (H1N1 vs H3N2), the co-circulation of influenza B strains and the number of antigenic strains. The pandemic waves were more remarkable for the number of hours lost than the number of employees taking time off work. Estimates of the seasonal absenteeism rate attributable to influenza and proportion of hours lost due to influenza infection were based on separate models, though a strong association between these estimates is evident.

The annual estimates of absenteeism and proportion of hours lost were correlated, though rates varied significantly from season to season (Figure 1). In 50% of the seasons, absenteeism ranged from 7%-15%, representing 0.07% to 0.11% of hours worked annually (inter-quartile range). The corresponding figures for the 10^th ^and 90^th ^percentiles are 5%-20% and 0.05%-0.13%, respectively. As estimates for the 2005/06 (A/California/7/2004) season were not statistically significant, the full range is uncertain and the minimum absenteeism rate may be significantly lower than 5%. Higher rates were associated with seasons where more than one distinct antigenic strain circulated. This correlation reflects a consistency of the estimates, as the impact of influenza on absenteeism and hours lost was estimated separately for each season. The pandemic waves were more remarkable for the hours lost than the number of employees taking time off from work (Figure 1). Employees who took time off due to a seasonal influenza infection took an average of 14 hours off per absence. By comparison, the average absence was 25 hours for the pandemic strain. These estimates are equivalent to the loss of approximately 20 days per 100 full time employees during a typical influenza season for a partially vaccinated population and 40 days during the pandemic period. For comparison purposes, all rates were prorated to an annual basis.

### By age and other employment characteristics

**Figure 2:**
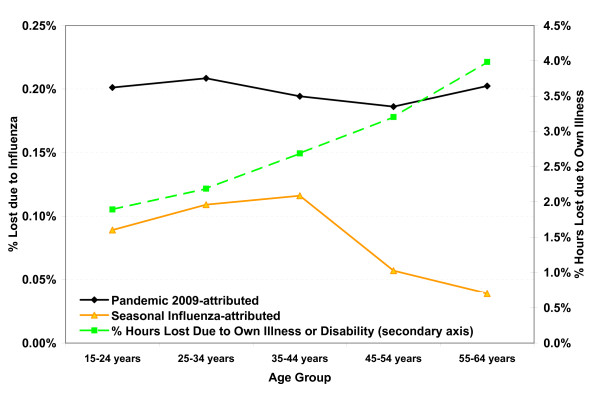
**Percent of potential hours worked annually that were lost due to influenza illness by age group**. The dashed line shows the increase in the proportion of hours lost due to own illness or disability with increasing age. The estimated proportion of hours lost due to an infection with the pandemic strain was similar for all age groups.

**Figure 3:**
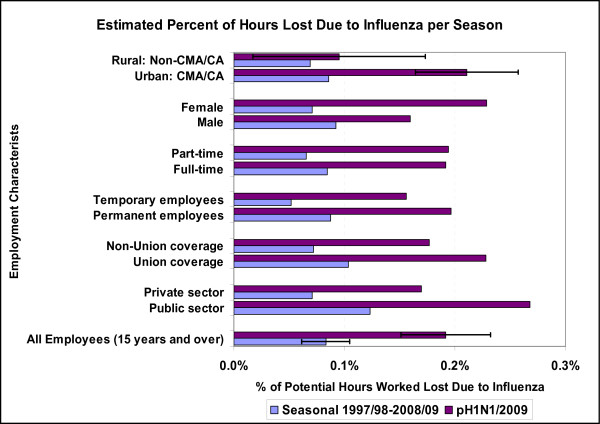
**Estimated percent of potential hours worked that were lost due to influenza per season**. The proportion of potential hours worked annually that were lost due to influenza over the two pandemic waves was 0.19% (95% CI 0.15-0.23) compared to 0.08% (0.06-0.10) for seasonal influenza. Confidence intervals were estimated based on the coefficient of variation of the corresponding parameter for the proxy variable for influenza activity and includes excess variation estimated by the inclusion of a dispersion parameter. As a result, the CIs were quite broad and differences by employment characteristics were not statistically significant. The CIs for the Urban/Rural split were included to illustrate. While, the proportion of hours lost varied significantly with the specific employment characteristics, these employment characteristics had less impact on hours lost due to influenza.

The proportion of hours lost due to one's own illness or disability increases significantly with age (Figure 2, secondary axis) [22], however, the proportion attributable to influenza was similar across age groups for the H1N1/2009 virus, though the statistical power to detect age-specific differences was poor. The level of influenza B activity was significantly associated with hours lost in the younger age groups only, which accounts for higher estimated rates among younger workers for seasonal influenza. Specific employment characteristics such as union status, job permanency, full or part time employment or a public or private sector employer had limited impact on hours lost due to influenza despite significant differences in absenteeism rates and hours lost for all illnesses or disability by age and employment characteristics [22]. The model estimates of the differences in hours lost due to influenza by employment characteristics, illustrated in Figure 3, were not statistically significant (after accounting for dispersion).

### Model Fit

**Figure 4:**
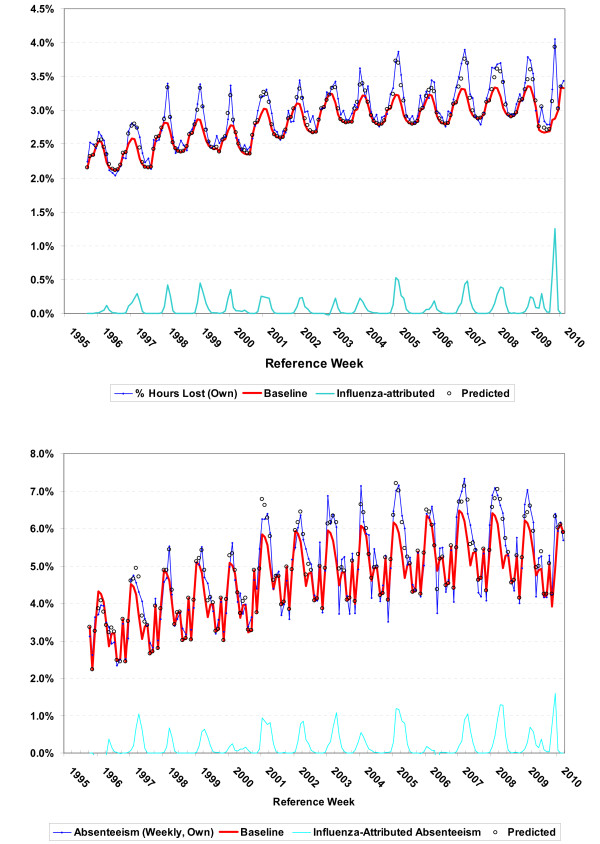
**Model fit showing the estimated baseline and attribution to influenza**. The actual data, a) the proportion of hours lost, and b) absenteeism rates are plotted against the reference week along with the model predicted values, the model estimated baseline and the attribution to influenza. Influenza is responsible for much of the seasonal variation and contributes significantly to peak absenteeism rates.

**Figure 5:**
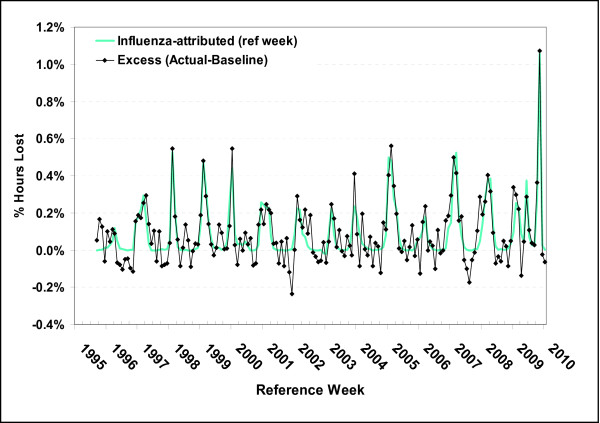
**Comparison of the attribution of hours lost to influenza: assessing model fit**. The actual hours lost less baseline (excess) is compared with the model predicted hours lost less baseline. The difference between the two curves are known as model residuals (and equal to actual - baseline). Residuals represent the variation not explained by the model. The influenza-attributed curve is smoother as the residuals, or unexplained variation, are not included in this time series. The residuals will average out over a season. The model fit is reasonable, though the model seems to miss the occasional dip in hours lost over the summer period.

**Figure 6:**
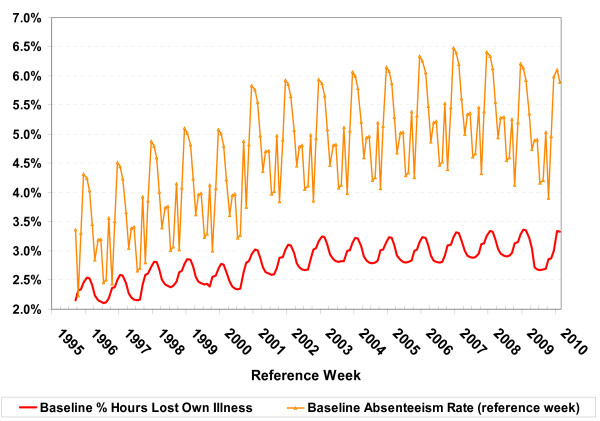
**A Comparison of the seasonal baseline for absenteeism rates and percent of potential hours worked that were lost due to own illness or disability**. The seasonal baselines in the absence of influenza activity for the two measures of time off work: absenteeism rate and hours lost due to own illness or disability, were estimated statistically. The baseline curves are distinct, with considerably more seasonal variation in the absenteeism rate than in the proportion of hours lost due to own illness or disability.

The overall model fit is shown in Figure 4 where the proportion of hours lost (a) and absenteeism rates (b) for the reference week are plotted along with their model predicted values, the model estimated baseline and the attribution to influenza. The model fit is shown in finer detail in Figure 5 where excess hours lost (actual - baseline) is compared to the attribution to influenza (predicted-baseline). The difference between the two curves are known as model residuals (and equal to actual - predicted), or the variation not explained by the model. The model fit is reasonable, though the model seems to miss the occasional dip in hours lost over the summer period (Figure 5). In Figure 6, a comparison of the two baseline curves shows strong seasonal variation and significant differences in the seasonal pattern of absenteeism and hours lost. The seasonal baseline for absenteeism rates has a much more pronounced seasonal variation with major peaks in the winter months and minor peaks in May, June and September. The minor peaks are not evident in the seasonal baseline curve for lost hours. Elsewhere these months have been associated with increased asthma admissions [23].

The expectation was that β_5 _(change in behavior during the pandemic) would be positive due to intensive public health messaging during the pandemic period reminding the public to stay home if sick. The β_5 _parameter was actually negative in the hours lost model, and not significant in the absenteeism model. This can be confirmed visually in Figure 4 where the proportion of hours lost is lower in the summer months of 2009 than it had been for many years.

## Discussion

This study confirms that estimates of absenteeism due to seasonal influenza typically ranged from 5% to 20%; higher absenteeism rates were associated with mixed seasons. These results are in reasonable agreement with general assumptions on the clinical attack rate for influenza, though it is noted that not everyone with symptoms consistent with an influenza like illness (ILI) [15,24] (sore throat, fever and cough are the most frequent symptoms) would necessarily take time off work. Symptoms can be mild for some; not everyone experiences a fever and many infections are believed to be asymptomatic [25,26]. Absenteeism rates for the 2009 pandemic were similar to those occurring for seasonal influenza. Employees, however, took more time off due to the pandemic strain than was typical for seasonal influenza. Employment characteristics had less impact on hours lost due to influenza than on the total hours lost for all illness and disability (Figure 2) [22].

Four special questions were added to the labour force survey in December 2009 through to February 2010 in order to estimate the impact of influenza on hours worked. Labour force survey participants were asked how many hours they took off work as a result of the 'flu' in the previous month due to their own illness as well as for the care of others. An estimated 9.0%, 4.4% and 3.5% of employed people were absent from work as a result of the 'flu' for November 2009, December 2009, and January 2010 respectively [27,28]. Workers also reported working additional hours due to the flu. In comparison, in this study we estimated an absenteeism rate due to influenza of 6.7% and 0.3% for the months of November and December 2009 respectively, and a negligible amount for January 2010 (Table 1). The two estimates, though both based on LFS participants, are different. Because of sample rotation, only about 5 out of 6 households surveyed in the November panel participated in the December survey. The November panel was asked about absences during the November reference week, while the December panel was asked about absences during the December reference week and about flu related absences during the whole month of November. Estimates of influenza-attributed absenteeism for the non-reference weeks were calculated based on the weekly level of influenza activity. The survey estimate from the four special questions included hours lost due to the respondent's own flu-related illness, care for others, and any flu-related medical appointments. However, the assessment that influenza-like symptoms were due to flu was at the respondents' discretion. There are many viruses that cause influenza like symptoms, and most 'flu' symptoms in December and January were most likely due to other viruses. After accounting for the differences in definitions, the two estimates of absenteeism for the month of November appear to be consistent. Our estimate of the average number of hours lost per absence was slightly higher than the estimate of hours lost due to 'flu' from the special survey (25 hours compared to 20 hours for 'flu'), and again this difference was possibly due to the potential inclusion of other ILI by the respondents in the special survey.

Economic studies of the benefits of influenza vaccination programs in the workplace avoid the costly process of directly measuring absenteeism due to influenza by comparing the number of days lost due to ILI in vaccinated and unvaccinated workers [29]. Confirmation of an influenza infection is possible through laboratory testing; however, this limits potential studies to a small population. The advantage of our approach is that the absenteeism estimates are specific to influenza, can be generalized to the Canadian labour force and include many seasons; however, the indirect estimation of hours lost due to influenza has other limitations, including the relatively large confidence intervals for sub-populations. This statistical approach has been used to estimate other characteristics of the disease burden attributable to influenza on the Canadian population, such as hospitalization [1,17] and mortality rates [6,8,16]. In comparison, the LFS is a relatively small sample of employed persons in Canada, with a data point available for only one week per month. As a result, this study did not have sufficient statistical power to assess the relative effects of the various employment characteristics on absenteeism rates, though the studies mentioned above were able to provide estimates for sub-populations with more precision. Proxy variables for the level of activity of other respiratory viruses such as parainfluenza and respiratory syncytial virus (RSV) were initially included in the model, but were dropped due to lack of statistical significance and because, based on these previous modelling experiences, it is reasonable to assume that hours lost due to other, non-influenza ILI would be captured in the seasonal baseline.

The inclusion of a scale parameter in the model inflated the confidence intervals of the estimated parameters, so it is unlikely that the level of statistical significance is overstated, however, the less than ideal model fit still suggests caution in the interpretation of model results. As this is a population-level study design, other explanations than those included in the model may be possible. The effect of public health messaging advising the public to stay home if sick is uncertain, as the proportion of hours lost was actually lower in the summer months of 2009 than for previous years. Would employees have taken less time off for other reasons in anticipation of possibly needing additional sick days due to a future infection with the pandemic strain? Absenteeism rates were not statistically significant for all seasons; it is not clear whether the lower peak absenteeism rates for the 2005/06 season were due to limited illnesses related to influenza that season as the model suggests, or due to other causes not included in the model. The robustness of annual estimates of disease burden is known to be less than ideal.

Despite higher vaccination coverage in recent years (increasing from approximately 10 to 25% of the working age Canadian population [30]), a slight upward trend in absenteeism rates due to seasonal influenza was noted for recent years (not shown). As the four seasons where a single antigenic strain dominated occurred early in the study period (1997/98, 1999/00, 2002/03 and 2003/04) and these seasons were associated with relatively low absenteeism (Figure 1), the apparent lack of association between vaccination coverage and absenteeism could to be explained by higher overall attack rates associated with the co-circulation of multiple influenza strains in recent years or, perhaps, increasing social pressures for self-isolation at home when sick.

## Conclusions

These estimates of absenteeism and the range of year-to-year variation should be a valuable contribution to the study of the economic burden of influenza and of potential use to cost-benefit analyses of workplace vaccination programs. At the community level, 50% of the cases were found to occur within a 4 to 5 week period [31], so, unlike other reasons for absenteeism, time off due to influenza illness is concentrated over a relatively short period time and is responsible for peak absenteeism rates.

## Abbreviations

95% CI: 95% confidence interval; ILI: influenza-like illness; LFS: Labour Force Survey; PHAC: Public Health Agency of Canada; RVS: Respiratory Syncytial Virus

## Competing interests

The authors declare that they have no competing interests.

## Authors' contributions

DS conceived the study, performed the analysis and drafted the manuscript. DS, HZ and JG contributed to the study design. HZ and JG contributed to the interpretation of study results. All authors revised the manuscript critically, and all approved the final version that was submitted.

## Pre-publication history

The pre-publication history for this paper can be accessed here:

http://www.biomedcentral.com/1471-2334/11/90/prepub
